# Supplementary figures and images for: Phlorizin Protects Against Oxidative Stress and Inflammation in Age-Related Macular Degeneration Model
Source: Biomolecules. 2025 Apr 3;15(4):523. doi: 10.3390/biom15040523 (PMC12025036; doi:10.3390/biom15040523)

Figure 3A

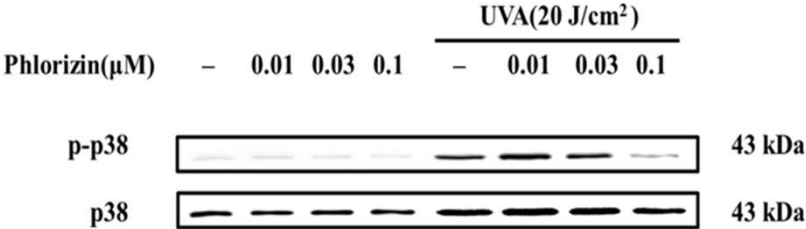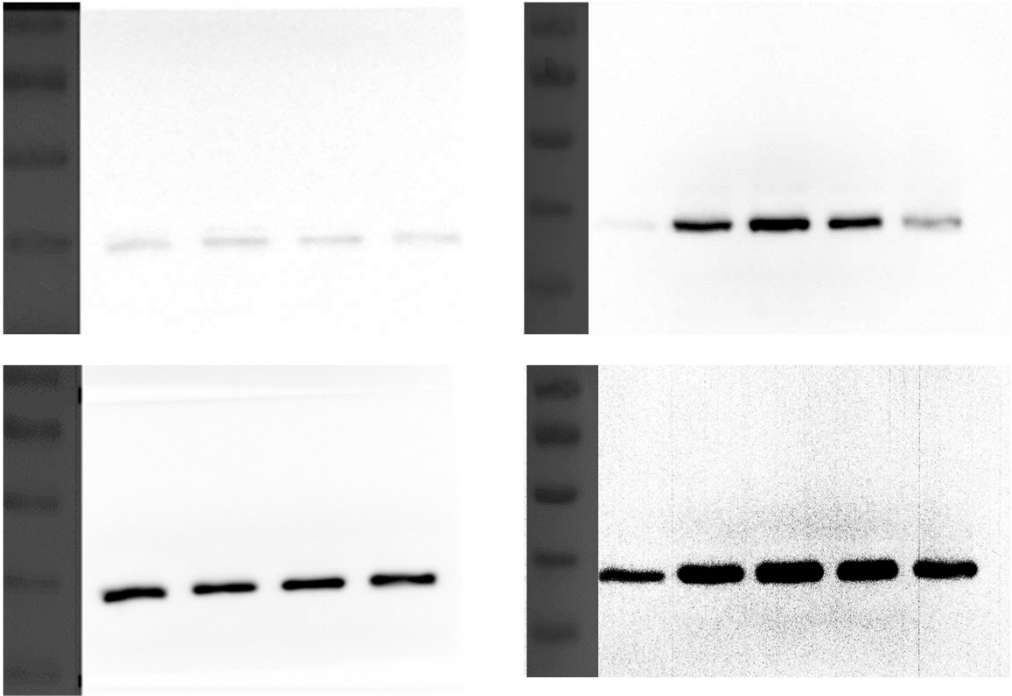

Figure 3B

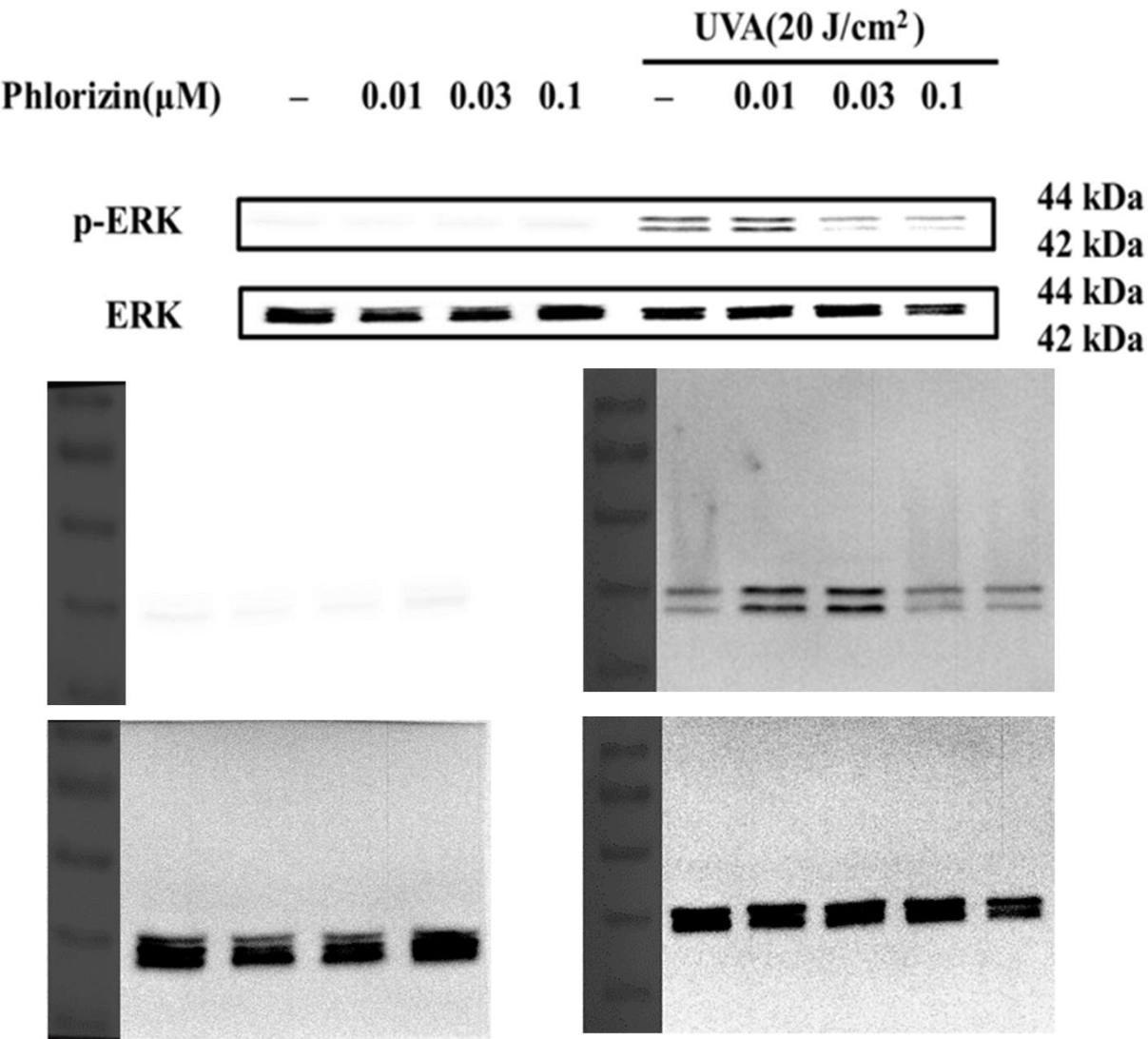

Figure 3C

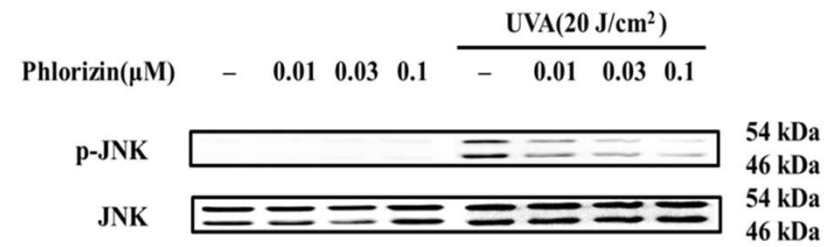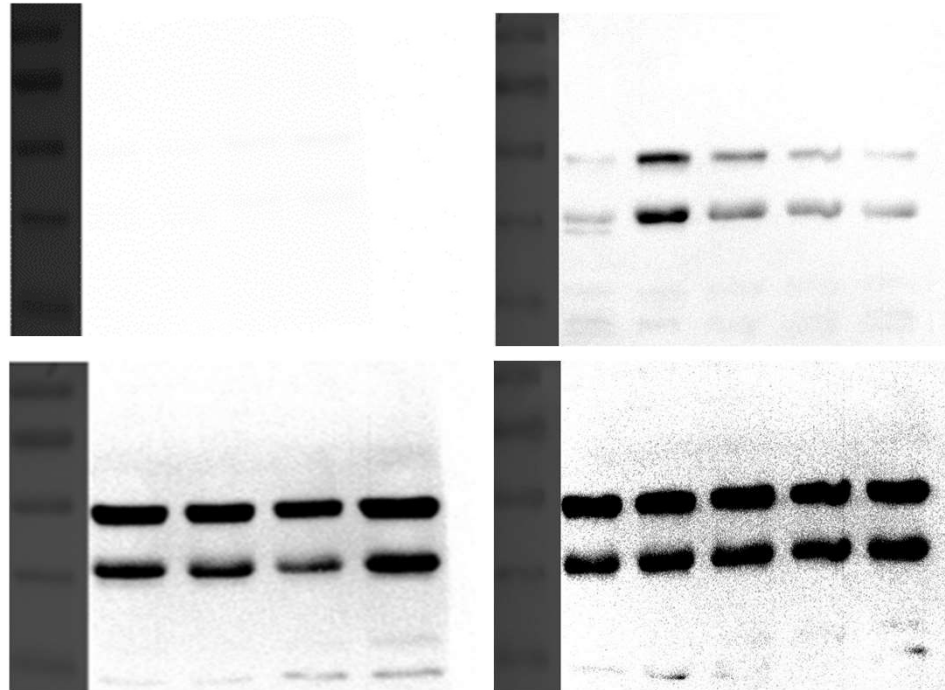

Figure 4A

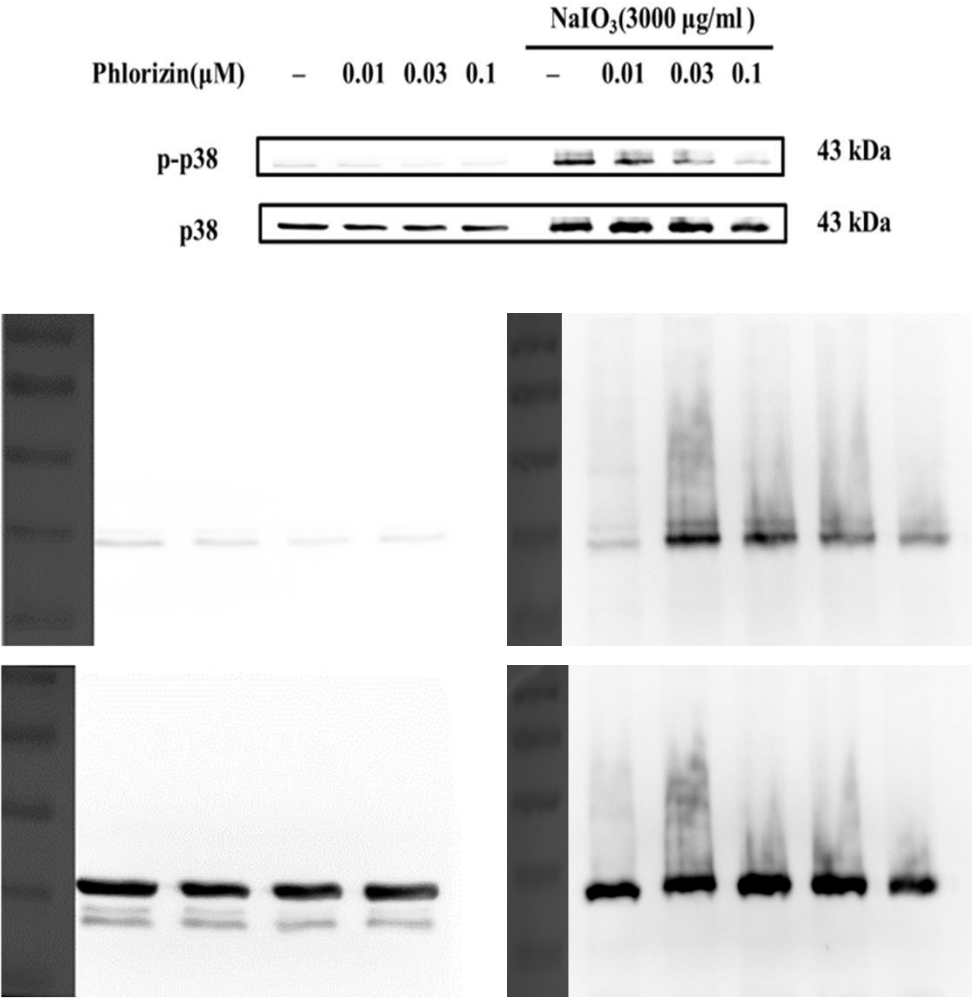

Figure 4B

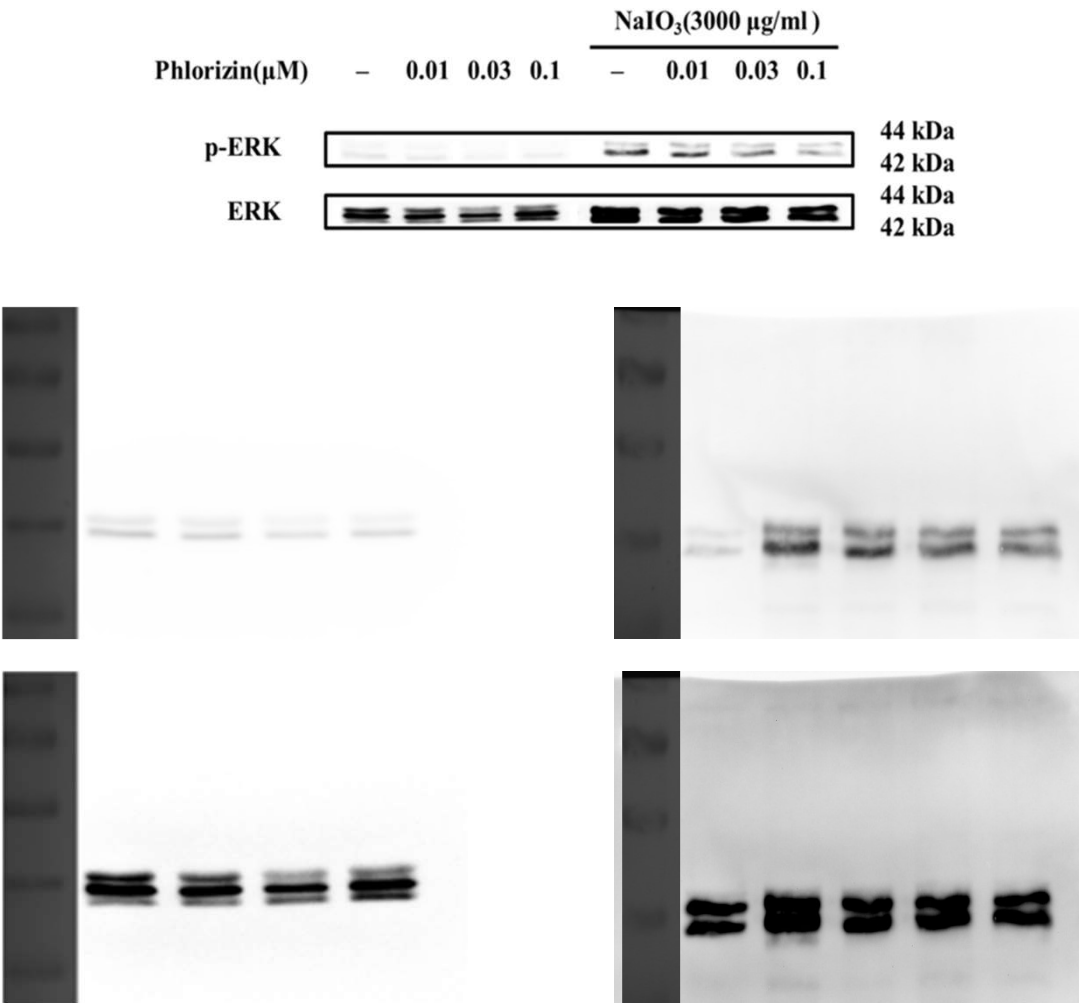

Figure 4C

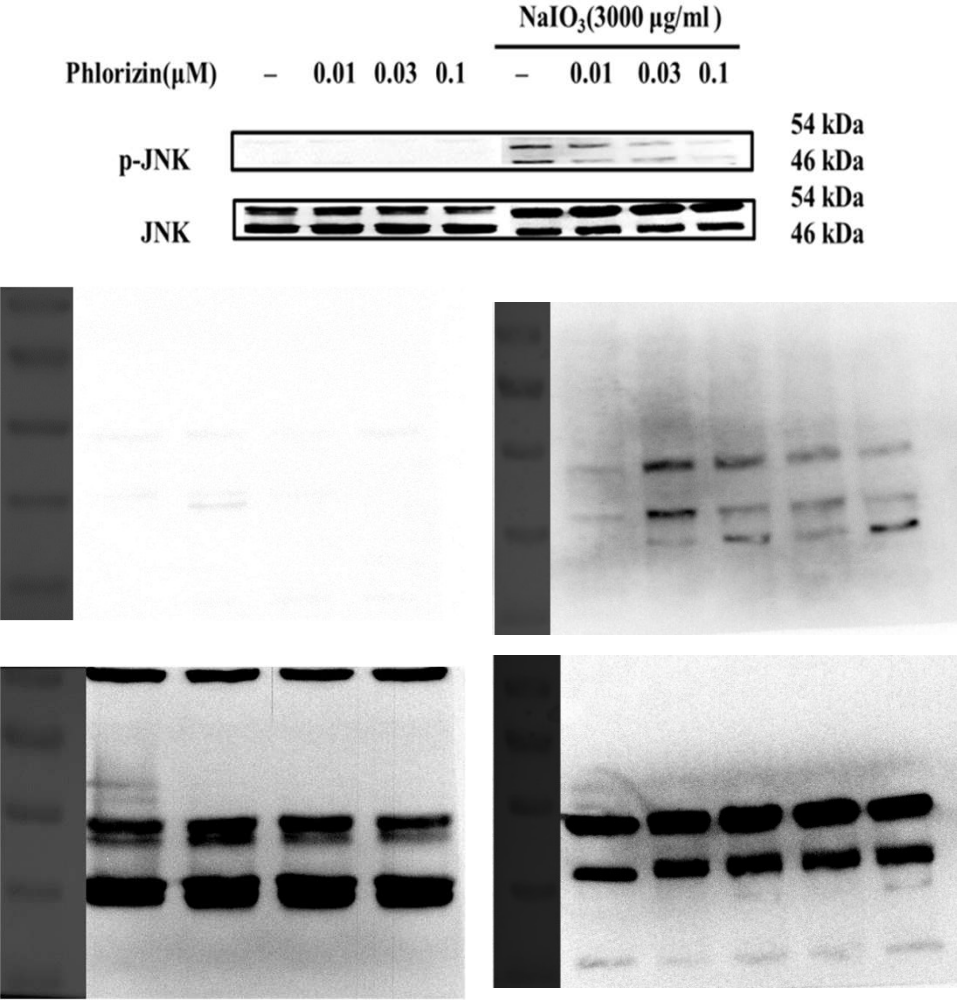

Figure 5A

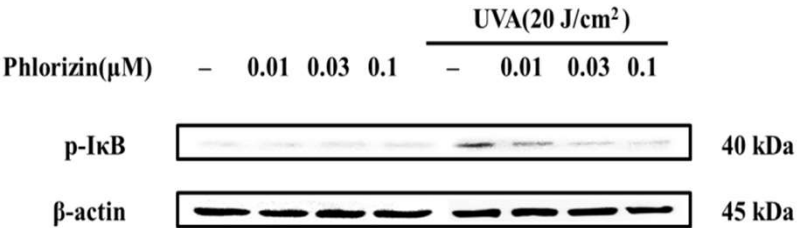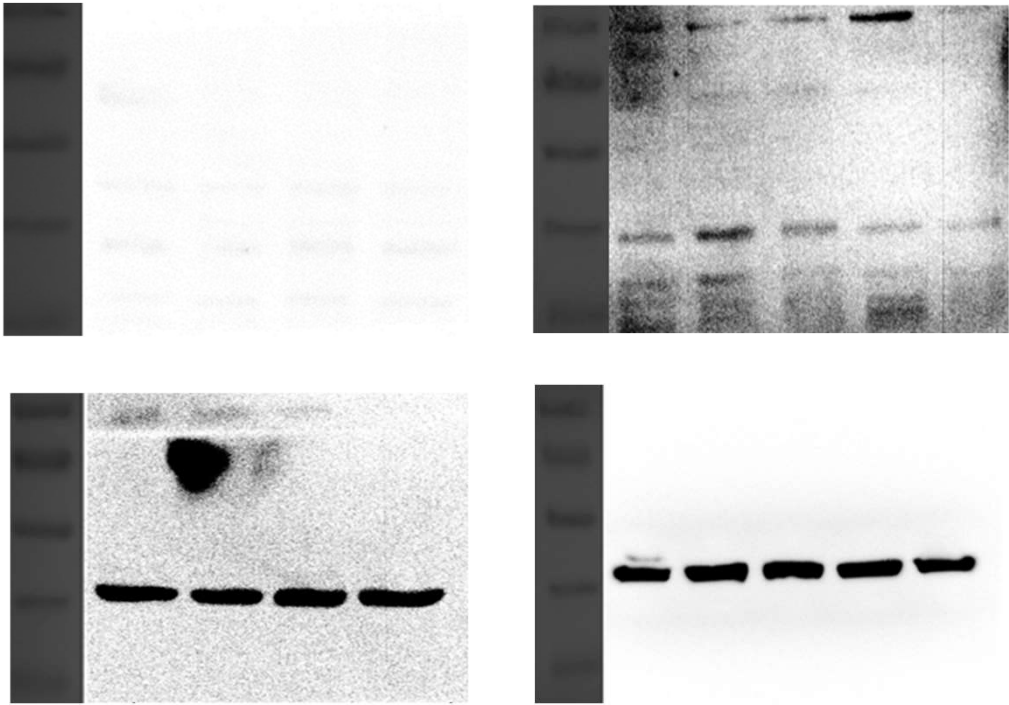

Figure 5B

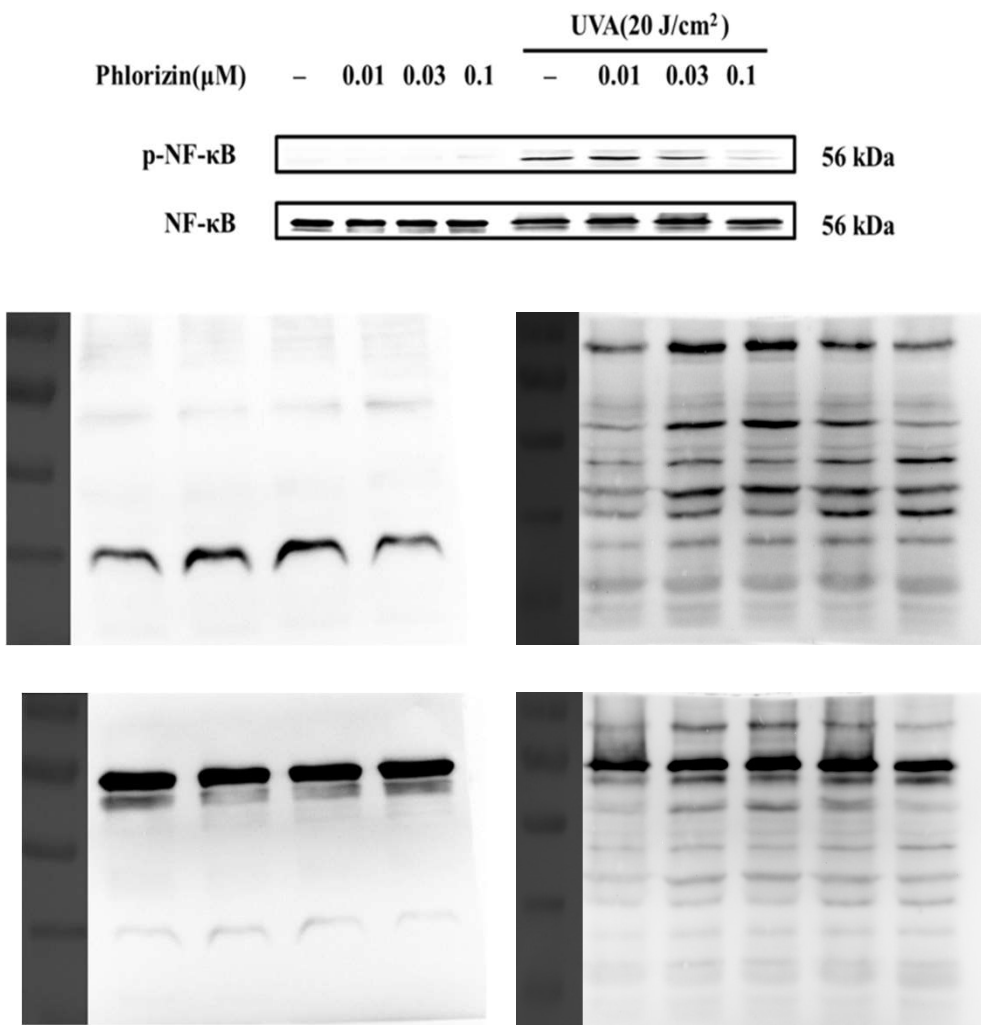

Figure 6A

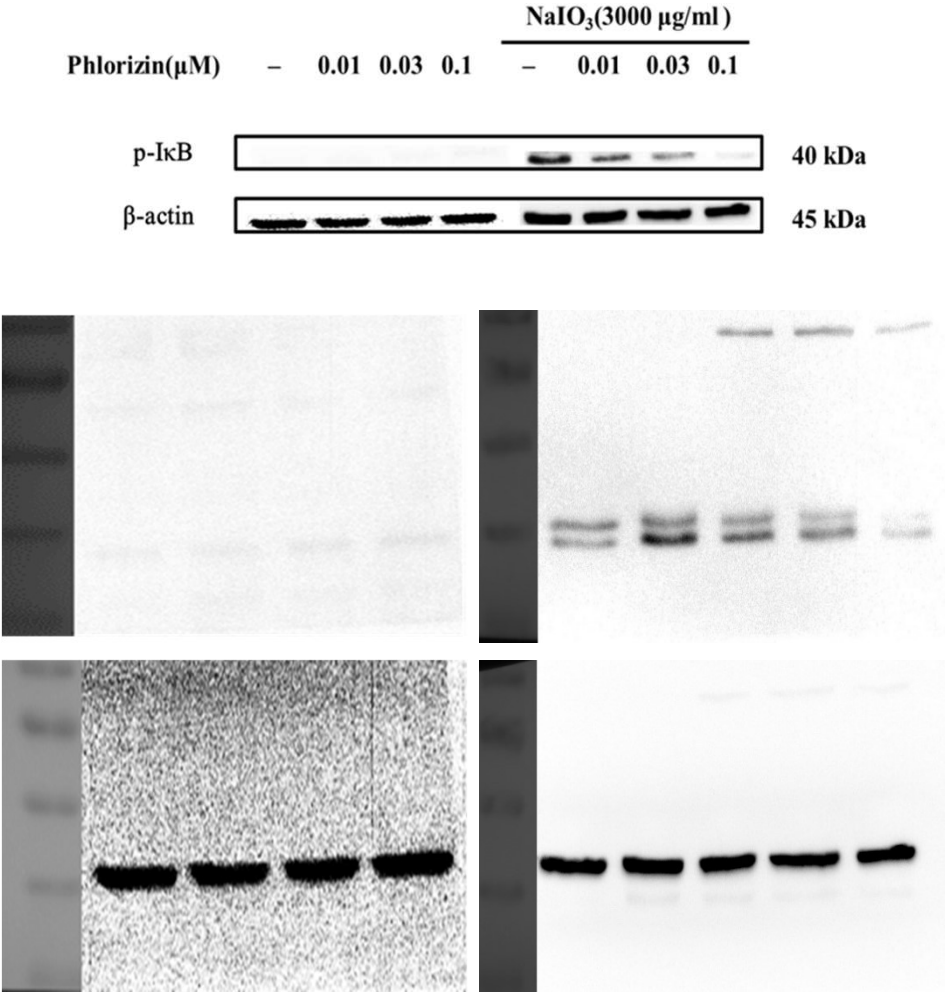

Figure 6B

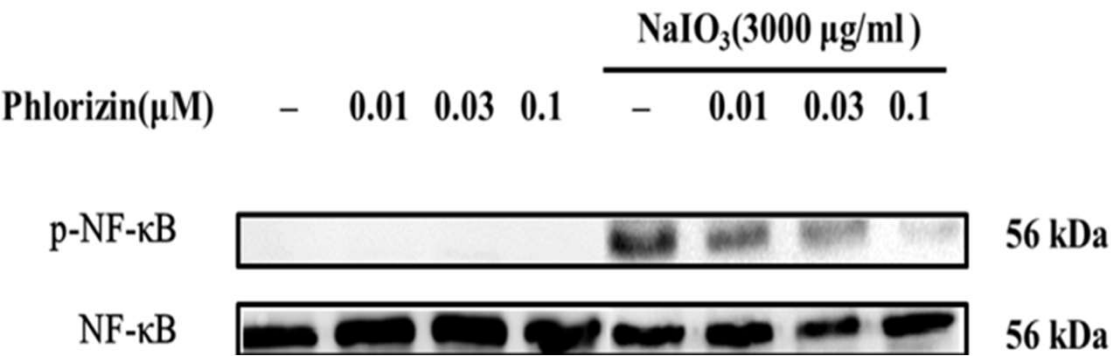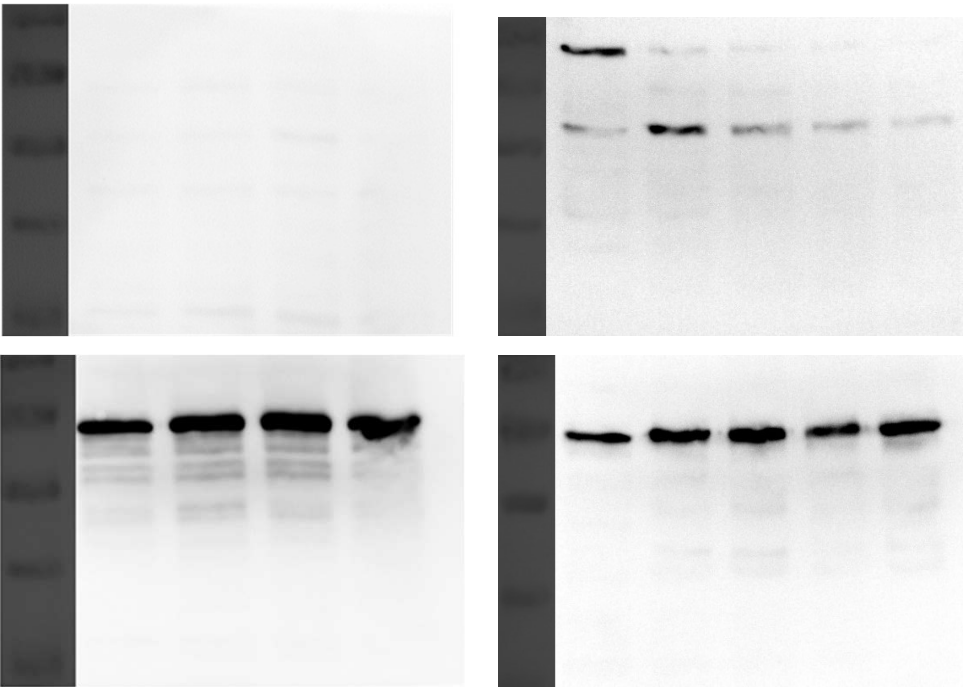

Supplement: Supplementary file 1 [file biomolecules-15-00523-s001.zip › biomolecules-3513531-supplementary.pdf]
